# Whooping Cough Statistics

**Published:** 1902-07-26

**Authors:** 


### Hospital Clinics and Medical Progress.

### WHOOPING COUGH STATISTICS.

There are few towns in which whooping cough is a notifiable disease. Aberdeen, however, obtained power, under its Corporation Act of 1881, to compel the notification by the medical attendant of all the principal infectious diseases inclusive of whooping cough, so that there now exists a complete record of all notified cases of that disease occurring in the city since the commencement of 1882. Moreover, in 1891 the Town Council adopted the Notification Act 1889, and added measles and whooping cough to the schedule. Thus dual notification of these maladies has been in operation since August 1891. In a paper prepared by the late Dr. James S. Laing [1] and since revised and extended by Professor Matthew Hay, M.D., the facts embodied in these returns have been carefully analysed and some interesting conclusions have been drawn from them. One curious and somewhat disheartening fact which is brought out is that neither the notification of whooping cough nor the measures consequent upon it, have led to any diminution of the prevalence of the disease. On the contrary there is an increase. There is however a certain diminution of the case-mortality.

A very striking point shown by the tables is the tendency of the disease to occur in epidemics, recurring at intervals of two years. Month by month during these 19 years whooping cough was always present, but every other year epidemics arose—generally, but by no means always, in the winter months, the highest peak of all in the curve having occurred about midsummer in 1898. It is curious to note that the curve of the case-mortality neither coincides with nor runs parallel with that of the attack-incidence. This has been before observed in regard to some other ailments, scarlet fever for example, the causes which influence prevalence of a disease being evidently different from those which influence its fatality.

From a clinical point of view perhaps the most important lesson taught by these tables is the preponderating influence of age in reference to case-mortality, the fatality of the disease being highest by far in the first and second years of life, and highest of all in the first quarter of the first year. In the case of any child, then, but especially in the case of a delicate child, it is worth while to take a very great deal of trouble to protect it from exposure to whooping cough infection until the first two years of its life are over. It is interesting again to notice that the case-mortality tends to increase as an epidemic progresses, being much higher towards the end of an epidemic than at its commencement, while it is usually highest of all in the intervals between epidemics, a point which we may perhaps paraphrase by saying that a child who has such a susceptibility to whooping cough as to catch it when there is not much infection about is likely to have it in a more serious form than one who only becomes affected when the infection is widespread.

To show the extent to which the fatality of the disease is influenced by social conditions a table has been prepared giving the case-mortality in houses of different sizes. The case-mortality in houses of one room is three-and-a-half times as great as in those containing five rooms and upwards.

An attempt has been made to compare some of the facts observed at Aberdeen with those recorded in regard to other towns, but in the absence of notification in most towns a complete comparison has not been possible. So far, however, as mortality affords a basis of comparison, it seems evident that the periodicity of the epidemic varies much in different towns, intervals of three years between successive epidemics being not infrequent. There seems no evidence of any atmospheric or similar wide-spread conditions determining the simultaneous occurrence of epidemics in various towns, the epidemicity appearing to be determined wholly by local conditions.

It is not without interest to inquire what use is made by the authorities of the information furnished to them by the system of notification which is in force in Aberdeen. Each case is visited by a sanitary officer who sees to its proper isolation, so far as it can be carried out with only a small percentage of removals to hospital, and leaves with the parents or guardians a printed set of instructions as to the precautions to be taken. Under section 57 of the Public Health (Scotland) Act, 1897, all the children of the family are prevented from attending school until the patient has recovered and a certificate has been granted by the medical attendant, or the medical officer of health, stating that the child or children desiring to return to school are "free from disease and infection, and that the house, and everything therein exposed to infection, has been disinfected." The disinfection of the house is always carried out by the sanitary staff, the infected bedding and clothing being removed to the disinfecting station for disinfection. In addition to this no known case of whooping cough in Aberdeen is permitted to move about outside so long as the patient is infectious, and prosecutions for such exposures have been undertaken. Poor little children! The case-mortality of those who live in one-roomed houses may well be high! And yet notwithstanding all these precautions the prevalence of the disease increases, while the mortality has only gone down a little—a very little—compared with other towns. Truly, of the eight towns mentioned, Aberdeen has nearly the lowest mortality from whooping cough, but on the other hand if one divides the whole period 1856-1899 into two portions, an early and a late, it appears that while Aberdeen shows an improvement in its whooping cough mortality of only two per 100,000, other towns show a far greater amelioration, the death-rate from this disease having fallen in Glasgow from 162 to 112, in Dundee from 121 to 72, and in Greenock from 133 to 65. Aberdeen started with a low mortality namely 65 per 100,000, and has a little more than held its own, its mortality having been reduced to 63. But these other towns have done far more. They have started with a high mortality which they have managed to vastly reduce. Hence, interesting as the figures are, it cannot be said that, taken by themselves, they say much for the system which is in vogue in the city of Aberdeen. Clearly some other influences are at work, to which these statistics give no clue.

[1] Public Health, July.

